# Electronic data capture – Narrowing the gap between clinical and data management

**DOI:** 10.4103/2229-3485.76282

**Published:** 2011

**Authors:** Deven Babre

**Affiliations:** *Data Management, PharmaNet Clinical Services Pvt. Ltd., Mumbai, Maharashtra, India*

Data collection in clinical trials is mainly a manual process; Investigators at the investigational sites manually transcribe/record data from the hospital files (source) on case report forms (CRFs). Clinical monitors from sponsor pharmaceutical companies or from Contract Research Organization (CRO) visit the investigational site to verify if the data transcribed/reported on CRF matches source data (hospital file).[1] Clinical monitors then collect verified CRFs and send the CRFs to clinical data management (CDM) team.

Clinical monitors use transmittal form to enable CDM team to ascertain the contents of shipment and the data in transmittal form match with respect to subject number, CRF included in the present shipment, any other documents added in the shipment and special comments/instruction if any, etc.

CDM team follows up with clinical monitors to get the observed discrepancies resolved within a predetermined turnaround time. Received CRFs are then marked as received, in the Clinical Data Management System (CDMS) to alert the data entry team to commence double data entry as per the established norms. Double data entry is considered as the first step of quality control (QC), as it ensures data entered is CDMS is accurate. After completion of double data entry,[1] data review/cleaning gets initiated by responsible members in CDM unit.

This process is laborious and time consuming as it involves time required by monitors to collect CRFs, time required by CDM team to perform double data entry, validation and raising query via Data Clarification Form (DCF). Sites sends resolved DCF back to CDM team. CDM team requires time to update/action in CDMS and also for QC updates/action to be taken.

This has direct/indirect impact on time for drug to come in market.[2] This mooted the idea of real-time data management, which, with technological innovation was not an impossible task. Hence, technology and innovation was used to its full extent, and Remote Data Capture (RDC) which is a synonym for Electronic Data Capture (EDC) was developed and implemented. EDC technology was expected to have real-time access for site personnel/investigator to enter/update data.[1] EDC technology was also expected to have real-time access for CDM team to perform data review/cleaning[3] and real-time alert for subject safety. Use of EDC technology was expected to improve efficiency and accuracy of data, speed up decision making process and reduce cost.[3]

For a data manager, to get resolution for a query in a traditional paper based study, took around 5–8 days, right from sending the query and getting the resolution signed from the site on DCF. The cost involved in this per DCF was around US$ 80–120. Thus, it was not only a time consuming process but also a costly affair. With EDC technology, the resolution of an electronic DCF can be obtained in 15 minutes from when the query/DCF is visible for site/investigator. This is very effective and less time consuming.

Implementation of EDC has resulted in a reduction of paper consumption and load on clinical monitors to manage such huge volume of paper. It has also reduced the risk of loss/damage of CRFs during transit and also the courier cost associated with transferring CRFs.

It is due to these reasons that EDC is preferred over traditional method. However, there are also some hurdles/obstacles which need proper attention. Vendors developing EDC software are continuing their efforts and are constantly upgrading capabilities by adding/deleting some features of EDC software. EDC software must be compliant with regulatory norms, robust, dynamic and user-friendly. Vendors and Pharmaceutical companies got motivated after the introduction of Critical Path Initiative by the US FDA in March 2004. Vendors made sincere and complete efforts to build an EDC software, which will take into account some important features such as a) ensuring compliance with regulatory norms, b) preventing unauthorized access to data, c) providing appropriate tools/module based on the role of the individual involved in clinical study, d) having electronic signature and electronic record and e) creating inbuilt capability to detect and keep control on fraudulent data.

Software vendors and people from industry responsible for developing the software have played a role in charting work shift and role changes associated with change from paper to EDC. The extent of role changes with shift from paper to EDC are a) data entry task shifted to site personnel/investigator, b) data review/cleaning became a joint venture of site personnel/investigator, clinical monitor and CDM team, c) creating a need for having a trainer preferably in CDM to impact training functionality of EDC software, d) clinical monitors to perform source data verification which is a QC task, e) CDM members have to generate extra manual review listings and perform this task which is manual and f) clinical monitors or data management team to address/resolve technical issues faced by site personnel/investigators.

Good Clinical Practice (GCP) and 21 CFR Part 11 requires validation for a software system used for processing clinical trial data. This data may be used to be submitted to regulatory agencies to get approval or may be used for post marketing trials. Validation is a process beyond testing. System validation life cycle consists of (a) user requirement specifications, (b) functional specification, (c) design specifications, (d) implementation and (e) testing. Computer System Validation (CSV) Testing and documentation as per 21 CFR Part 11 compliance guidelines is required. Validation documentation may consist of user requirement specification, validation plan, functional requirements specification, system design specification, installation qualification, operational qualification and performance qualification, traceability matrix and validation summary report.

Software vendors successfully developed the technology of entering clinical data directly into the EDC software. However, in this situation, the site had no direct control over their source data, as they would have when data is collected using paper.[4] 21 CFR Part 11 regulation was a mandate that any EDC software must comply with. It commonly defines the criteria under which electronic records and electronic signatures are considered to be trustworthy, reliable and equivalent to paper records. Part 11 has requirements to implement controls, including audits, system validations, audit trails, electronic signature and documentation for software and systems involved in processing electronic data that are (a) required to be maintained by FDA rules and (b) demonstrate compliance to a predicated rule.

In EDC study, the investigator enters data and signs electronically for accuracy, reliability and completion of all data points. However, the investigator later can add, modify or delete data in the EDC system in future at any time-point, until the electronic CRF pages are locked and no more adding/updating the data is permitted. Investigator at this point should sign for the changes made to electronic CRF data points which were entered or changed. At the end all data entries, modifications or deletions must be signed for by the investigator, as he/she is owner of that clinical data. This is why signing by the investigator is so important.

General challenges/issues exist. Investigational sites personnel/investigators find data entry as tough/tedious task. Multiple EDC software has created difficulty/confusion to non-CDM members. Sponsor companies/CROs have to modify their SOP/instructions to accommodate functionality/features available in EDC software, upgrades/enhancements made to the EDC software and innovative techniques/methods developed by team members with increase in experience. There is a need to developing effective training for EDC software, which is study specific for a given protocol. The investigational sites/personnel are spread across the globe. Hence, they require 24 × 7 technical support and guidance. Also, in some cases, the support is expected in local language.

EDC, as a technology, is well developed, well accepted/practiced in industry. It is true that implementation of EDC has speeded up certain processes and improved the turnaround time for accessibility/updating data real time. However, few challenges still exist as researchers are trying to evaluate if quality of data produced by traditional paper based studies is better or equivalent compared to data generated by EDC. Paper is eliminated but using technology, e.g. internet, software EDC and other additional services such as call center, is it true that EDC still can be considered as a cost-effective solution?
